# Making Sense of a New Transport System: An Ethnographic Study of the Cambridgeshire Guided Busway

**DOI:** 10.1371/journal.pone.0069254

**Published:** 2013-07-30

**Authors:** Caroline H. D. Jones, Simon Cohn, David Ogilvie

**Affiliations:** 1 MRC Epidemiology Unit and UKCRC Centre for Diet and Activity Research, University of Cambridge, United Kingdom; 2 Institute of Public Health, University of Cambridge, Cambridge, United Kingdom; Edinburgh University, United Kingdom

## Abstract

An increase in public transport use has the potential to contribute to improving population health, and there is growing interest in innovative public transport systems. Yet how new public transport infrastructure is experienced and integrated (or not) into daily practice is little understood. We investigated how the Cambridgeshire Guided Busway, UK, was used and experienced in the weeks following its opening, using the method of participant observation (travelling on the busway and observing and talking to passengers) and drawing on Normalization Process Theory to interpret our data. Using excerpts of field notes to support our interpretations, we describe how the ease with which the new transport system could be integrated into existing daily routines was important in determining whether individuals would continue to use it. It emerged that there were two groups of passengers with different experiences and attitudes. Passengers who had previously travelled frequently on regular bus services did not perceive the new system to be an improvement; consequently, they were frustrated that it was differentiated from and not coherent with the regular system. In contrast, passengers who had previously travelled almost exclusively by car appraised the busway positively and perceived it to be a novel and superior form of travel. Our rich qualitative account highlights the varied and creative ways in which people learn to use new public transport and integrate it into their everyday lives. This has consequences for the introduction and promotion of future transport innovations. It is important to emphasise the novelty of new public transport, but also the ways in which its use can become ordinary and routine. Addressing these issues could help to promote uptake of other public transport interventions, which may contribute to increasing physical activity and improving population health.

## Introduction

Increased use of public transport (and a corresponding reduction in car use) has the potential to contribute to achieving a variety of goals in environmental, health and transport policy. Yet what happens when new public transport infrastructure is introduced, and how it is experienced and integrated (or not) into daily practice, is little understood. This ethnographic account documents the experience and use of new transport infrastructure in Cambridgeshire, UK, during the weeks following its opening. Data were collected by observing and speaking to passengers travelling or waiting for buses on the Cambridgeshire Guided Busway. In documenting the ways that people initially responded to the introduction of the busway, we attempt to understand how changes in infrastructure become embedded in everyday social practice. Our focus is on the ‘micro-level’ experiences of individuals and small groups of people who found themselves adopting and adapting to the innovation; and on how they engaged dynamically with the new infrastructure, rather than being passive recipients of it.

We are particularly interested in the potential for the Cambridgeshire Guided Busway and other transport innovations to become integrated into social practice from a public health perspective. A population shift from using private motor vehicles towards greater use of public transport has the potential to contribute to population health improvement. Compared with car travel, use of public transport has been shown to be associated with greater walking and overall physical activity [1] [2] [3], which in turn enhance wellbeing and reduce the risk of diseases including cardiovascular disease, diabetes and some cancers [4]. Furthermore, significant health benefits have been predicted to result from reducing the greenhouse gas emissions associated with the use of motor vehicles [5]. The introduction of a 9.6-mile light rail line in Charlotte, North Carolina was predicted to save $12.6 million in total healthcare costs over nine years [6].

In Great Britain, commuting to work is a large contributor to overall travel, accounting for 19% of the total distance travelled by individuals. While around 70% of commuting journeys are made by car, fewer than 10% are made by bus [7]. The existence of a large group of people with the potential to change their travel behaviour, coupled with evidence for substantial potential health benefits consequent on such a change, suggests that new transport systems might be regarded as public health interventions. It is therefore important to understand how people experience and make sense of them in order that their benefits might be fully realized.

### The Busway

The city of Cambridge (population 108,000) is connected to its surrounding smaller towns and villages by a good road network. In Cambridge, the 2011 Travel for Work Survey reported that 5% of respondents travelled to work by bus [8]. While the proportion of commuters travelling by car is, at 56% [8], lower than the UK average of around 70% [7], the cost of housing in the city is relatively high and there is limited public transport serving the surrounding predominantly rural area. As a result, there are relatively high levels of rural car ownership [9].

**Figure 1:**
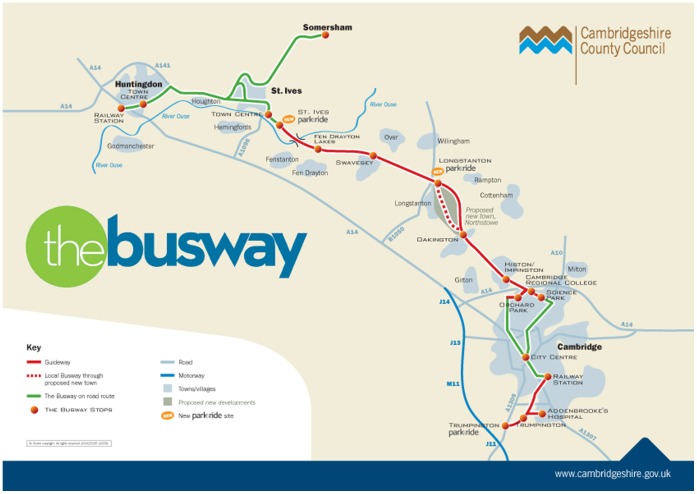
The Cambridgeshire Guided Busway. Reproduced with permission from Cambridgeshire County Council and Stagecoach. The guideway runs from St Ives to the northern edge of Cambridge city, and from Cambridge railway station south to Addenbrooke’s Hospital and Trumpington. Through the city centre, and north of St Ives, guided buses join the road network.

The Cambridgeshire Guided Busway (known as “the busway”) is a major new piece of transport infrastructure linking Cambridge city centre with surrounding towns and villages [10]. Buses run on a segregated track that mostly follows a disused railway line. The guideway runs from St Ives (a town to the northwest of Cambridge) past smaller towns and settlements to the outskirts of Cambridge, where buses join the road network through the city centre to the railway station. From there, another section of guideway runs south to Addenbrooke’s Hospital and on to Trumpington, a suburb of Cambridge (see Figure 1). The buses are fitted with guide wheels that guide the bus between the kerbs of the guideway, which is a track consisting of concrete beams. The driver does not need to steer, the journey is smooth, and buses run on a dedicated route avoiding notoriously congested trunk roads. It was therefore claimed that the opening of the busway would lead to faster, more reliable, and more comfortable journeys [11]. There is also a traffic-free route for pedestrians, cyclists and horse-riders adjacent to the guideway; it is not the focus of this study.

The busway opened in 2011, two years later than originally announced, and around £64 million over budget [12]. Repeated delays and cost overruns resulted in a great deal of critical media coverage which had not subsided when buses began running on the busway on 7 August 2011. Over 600,000 journeys were made on the busway in its first three months of operation, 35% more than forecast [13], and the one millionth passenger boarded in early January 2012 [14].

This ethnographic account documents how the guided busway was used and experienced by passengers in those weeks following its opening. It exploits the opportunity to explore the period of introduction and “trialling” of a new transport intervention, and investigate whether and how it became embedded in everyday practice.

Our research forms part of a wider project, the Commuting and Health in Cambridge study, which aims to evaluate the effects of the busway on travel behaviour, physical activity and wider health impacts [15]. It includes a longitudinal cohort study and smaller embedded qualitative studies [16] [17]. Our ethnographic study involves a different group of participants to the main cohort, and draws on participant observation to capture the views and experiences of users as they travel. The aim is to provide insight into the initial experiences and use of an innovative piece of transport infrastructure, allowing us to observe and report on spontaneous reactions and how people make sense of, learn to use and evaluate the intervention, before it has become routine and ordinary.

Qualitative evaluation of interventions and more diffuse structural changes has become increasingly popular, since it enables researchers to understand the importance of context [18] and capture the perspectives of recipients, rather than being constrained by measures pre-defined by the researcher. Medical Research Council guidance reiterates the value of qualitative methods in the evaluation of population health interventions [19]. Thus, whilst qualitative methods have previously been applied to the study of public transport, and travel more generally [20] [21] [22], our study extends previous work by exploring new transport infrastructure from the perspective that it might function as an intervention likely to have a range of effects, including population health benefits.

### Normalization Process Theory

In order to provide a conceptual framework within which to interrogate our data, and so that we can suggest how our accounts of individual experiences might represent a wider set of responses across a population, we draw on Normalization Process Theory (NPT) [23]. NPT describes the ways in which innovations become embedded in everyday practice (normalized) across a number of different domains. It is a tool for understanding and explaining the overlapping processes relevant to the adoption and integration of something new, and has been used to evaluate complex interventions in health care settings [24] [25].

Core constructs from NPT which are particularly useful in interpreting our data are coherence and reflexive monitoring [23]. The former relates to the extent to which an innovation is experienced as a continuation or disruption of what has gone before. It includes how the innovation and related practices are regarded as different from previous ones; to what degree people have an understanding of the aims and benefits of the new practices and the specific tasks involved; and the extent to which the innovation is regarded as important. Reflexive monitoring refers to how people assess and understand the effects of a new innovation and related practices as they are introduced, and includes how participants collect and make sense of information about the innovation and its effectiveness. An important dimension of this is how people work as individuals or informal groups in making their evaluations. Overall, a central feature of this sociological theory which we emphasize in our analysis is the way in which aspects of an innovation may be subject to both individual and more collective activities in order to become successfully embedded.

From the perspective of NPT, we were able to address our aim of exploring people’s experiences and use of new transport infrastructure, focusing on whether and how it became integrated and normalized. This is important from a public health perspective because promoting a shift from car travel to public transport has the potential to increase physical activity and improve population health.

## Methods

Fieldwork took place following the opening of the busway, between September and December 2011. CJ – an experienced female social science and public health researcher – travelled extensively on the busway on weekdays throughout this period, at different times of day (morning, afternoon, early evening), observing and interacting with passengers at bus stops and on the guided buses themselves. Fieldwork took place on week days because most commuting journeys take place during the week and, as discussed above, there is potential to reduce the proportion of commuting journeys made by car and increase the proportion made by public transport. Sampling was opportunistic: CJ spoke to passengers she encountered on days that she travelled on the busway. She got on and off the guided buses at different bus-stops, and spoke to passengers at different points along the entire route. This method – participant observation – is inherently flexible and inductive so that the exact nature of interactions is not predetermined; it involves casual conversations, fleeting encounters and informal methods [26]. There was no formal topic guide; passengers were encouraged to discuss any aspects of their experience of the guided busway. However, we were particularly interested in travellers’ reasons for using the busway and how it fitted into their everyday lives, and so passengers were asked to expand on those issues. During fieldwork it emerged that travellers’ previous modes of travel were important, and so future fieldwork also encouraged participants to discuss how they had travelled before the busway opened.

Participant observation has a long tradition in the social sciences, and is a very established method used to capture aspects of everyday experience [27]. Frequently combined with informal interviews and conversations, as in this study, its chief strength is to contextualise what people say in terms of what they actually do. In order to confidently suggest wider representativeness, the general approach of participant observation does not rely on an a priori sampling strategy in order to justify the relevance of the empirical data collected. Rather than claiming that specific incidences observed or comments collected are directly generalisable, the strength of the approach lies in identifying underlying issues or tensions that are typical, and it is therefore at the level of the overall argument that relevance is claimed [28]. Whilst many have debated the extent to which participation might compete with the act of observing, this tension has itself proved highly productive [29].

Three core issues can be highlighted. The first is the overall commitment to reducing the distance between the observer and the observed, in order to approach seeing the world from another person’s point of view [30]. In addition, participation with others means that recording what people say is always done in the context of what they do, ensuring that overly-literal interpretation of interview data is avoided [30]. Finally, participant observation allows the personal experiences of the researcher to be a potentially valid source of experiential data, which can help interpret the views and actions of others [31]. Rather than attempting to encapsulate a complete and holistic picture of social interaction, we adopted a more particular and defined focus (in this case, use of the busway) over a relatively short period of time, an approach that has been described as “micro-ethnography” [32].

### Ethics Statement

CJ identified herself to all travellers she approached and engaged with. She wore an identification badge, explained the study to travellers to whom she spoke, and asked for their verbal consent to take part and for notes about conversations to be written. Passengers who spoke with the researcher were given a participant information sheet to take away which included contact details for the research team. As with much ethnographic research [26], obtaining written consent was neither appropriate nor feasible because many encounters were fleeting; it would have been inconvenient to fill in and sign a form; and the topic of travel behaviour is not likely to be sensitive. In all cases, no identifying information was recorded; travellers cannot be identified by appearance, name, or date and time of travel. Passengers who did not directly engage with the researcher are only part of the general backdrop of observations and as such, do not constitute ‘research participants’. Furthermore, none of the data are likely to be sensitive, and the researcher’s presence had little or no impact. Two of the three authors (CJ and SC) are experienced socials scientists; and the published guidelines of the Association for Social Anthropologists [26] were adhered to. The study and its consent procedures were approved by the Cambridge University Psychology Research Ethics Committee (reference number 2011.50). Fieldwork was carried out with the permission of Cambridgeshire County Council (the local authority responsible for the busway) and the relevant bus operators (Stagecoach and Whippet).

Conversations were not audio recorded since it was unfeasible in a busy public transport environment to prevent non-participants from being recorded without their consent. Instead, field notes were taken, excluding any identifiable information so that participants remained anonymous.

### Data Analysis

Data analysis and interpretation were inductive. Data collection and analysis were concurrent and emerging themes informed future data collection [32]. This grounded theory approach ensured that analysis was driven by the data, and interpretations always emerged from and were grounded in the data [33]. Data collection ceased when data saturation was reached, that is when new data no longer generated new themes. Codes were developed and segments of the field notes were assigned to these by CJ. Codes were then grouped into broader themes identified from patterns in the data. Interim descriptive accounts of the data and analysis were discussed between the authors throughout the fieldwork period, to guide further data collection and analysis and to validate the emerging findings. Data analysis was completed, and this manuscript was prepared in accordance with published reporting criteria for qualitative research [34] [35], in the five months following fieldwork. Data are held by the UKCRC Centre for Diet and Activity Research; the corresponding author can be contacted for more information.

## Results

Trips were taken by the fieldworker on 20 mornings and 21 afternoons or early evenings during the study period. During each of these trips, CJ spoke to multiple passengers, and observed others without speaking to them. Those passengers whom CJ approached were informed about the study, and all consented to field notes being taken and being included in the study (100% participation rate). In discussion with the wider research team, it was agreed that data saturation had been reached after these trips and so fieldwork was discontinued. We present our results according to three main overarching themes, and using excerpts of field notes to support our interpretations.

### Early Experiences of the Busway

Forming an understanding of the purpose, benefits and value of the busway upon using it for the first time was clearly important for travellers in determining their future use. This was related to the extent to which the busway was normalized and became a routine part of people’s working day.

Early experiences were important in determining ease of use and compatibility with existing practices. The ease with which the busway could be integrated into existing daily routines was significant:

*I sat next to a man on the bus. [He said that the first time he used the busway he] was going out after work for some drinks and he didn’t want to drive. So he got a lift to the busway, took the busway to work, got the busway back after the drinks, and got a taxi from St Ives bus stop to home. He did it that time so he could drink. But it worked out well, it was easy, so he decided to use the busway more. So he’s been going on it to work.*

Hence early experiences in which the busway was perceived to be convenient and easy to use led to continued use and contributed to normalization.

However, for many the busway had not yet become embedded within the study period. For example, the issue of ticketing proved a major source of confusion:

*[A lady got on the bus and sat next to me.] When she first got on the bus she was unhappy about it – she said they couldn’t work the tickets out, it was a faff and they probably shouldn’t have bothered. They bought Whippet tickets, then the next Whippet bus wasn’t for an hour and they couldn’t wait that long, so they’d had to buy another, Stagecoach, ticket. Two buses had gone past while they were at the ticket machine and hadn’t waited for them to get on.*

Confusion regarding ticketing largely stemmed from the fact that two bus companies operate on the busway: Stagecoach (a national bus company) and Go Whippet (a family company local to Cambridgeshire). The buses are almost identical, and the services differ only in their routes across the city, and in whether they continue south to Trumpington, or continue north of St Ives. Yet tickets purchased from one bus company cannot be used on the other operator’s buses, regardless of which is the first bus to arrive. This was confusing and frustrating for passengers, especially those who were not familiar with the different bus companies operating.

Where ticket machines are available, passengers are requested to buy their tickets before boarding the bus (this is atypical for the UK, where most bus operators require passengers to purchase tickets on board). Not all passengers did so; in response, some drivers allowed people to buy tickets upon boarding, which was then a cause for complaint by some regular passengers whose journey was delayed. Other drivers chose not to wait, leaving surprised passengers to buy their ticket from a machine and wait for the next bus. These types of negative early experiences delayed and perhaps prevented the busway from becoming normalized and integrated into travellers’ daily practice.

### Collective Learning

Initially many passengers perceived that the busway was a novel system, which would thus require experience and learning:

*A group of 4–5 people (aged around 60 years) were standing together near the bus stop discussing busway tickets and routes. One of the women went to look at the information displayed on the bus stop; she came back and told the rest of the group that they could have got on the previous bus after all. She hadn’t realised it would have stopped where they’d wanted. “You live and learn,” she said.*

As demonstrated by this example, passengers sometimes learned how to operationalize the busway collectively. Communal learning extended to information being offered by strangers and bus drivers, who can be thought of as key participants in driving the innovation forward:

*When we got to a bus stop in town, the driver turned around and said “This is the last city centre stop”. Some people at the back didn’t hear, so a passenger near the front repeated the message down the bus. No-one got off and we carried on.*

*There were three women in front of me on the bus (aged around 30 years-old). They were studying the busway brochure detailing the route and timetable: they’d never used it before. A man on the seat opposite leaned over and told them that you can’t travel on a Whippet bus with a Stagecoach ticket. They said thank you.*

Thus passengers and drivers helped each other to make sense of how to use the busway, and operationalize the practices associated with it.

### Two Distinct Passenger Groups

A third theme served to discriminate two distinct passenger groups, as introduced by the following account:

*There were two adults sitting on the bench at the bus stop as I approached. One was a woman (around 30 years-old) and the other was a man (around 30–40 years-old): they didn’t know each other and they weren’t travelling together. Both took the bus from St Ives to Cambridge city centre each day for work. When I asked them about the busway, the woman rolled her eyes. She said it’d made absolutely no difference to her journey. She used to get the regular bus: that old service was better because it was quicker and wasn’t as busy. She had nothing positive to say about the new busway. The man, however, was very positive about it. He used to drive to work. He said that the car journey could take a long time and wasn’t as relaxing; also, since he drives all day for work he described how he enjoys now not having to drive to and from work as well.*

It is striking in the account above that the two passengers took the same service each day, yet had very different experiences and opinions. During fieldwork it quickly emerged that there were two distinct groups of travellers who experienced the busway differently: those who prior to the opening of the busway had travelled regularly by bus, and those who had mainly travelled by car.

Previous bus users and previous car users articulated differences in perceptions of coherence of the new service in relation to the conventional bus network, and in evaluation of it. The former had regularly used public transport prior to the opening of the busway; in many cases, their previous service had now been discontinued. They tended not to describe the busway positively; from their perspective it was not an obvious improvement and in some cases was felt to be worse: *“it actually takes longer because it stops at more stops along the way”; “the bus gets really crowded and noisy”*. Previous bus users were disappointed that the busway was not superior to the regular service, or was in fact inferior – *“for people like me, who used to have a good bus service, it’s frustrating that now it’s slower and you can’t always get a seat”*.

In contrast, those who had previously been almost exclusively car users described the new service positively in comparison to the car, and attributed greater worth to it*: “it’s cheaper than driving to work”; “I can sit on the bus and relax, not worry about the traffic”; “it’s easier, more convenient”.* These passengers appeared to be experiencing the benefits of public transport in general for the first time. Many of their positive remarks might have been applied to other forms of public transport (such as regular bus or rail travel) and were not specific to the busway; for example, not having to concentrate on driving, and the reduced cost of travel.

Nevertheless, some of these passengers positively discriminated between the busway and regular public transport, even if they had little experience of the latter – for example saying they *“wouldn’t use other public transport”* or *“I’m not a public transport user”*.

As previous bus users got into the routine of using the busway they rapidly began to perceive it simply as an extension of other public transport systems, as the following except from the field notes captures:

*One early evening I was waiting amongst a group of other passengers at a bus stop at Addenbrooke’s Hospital to get a guided bus towards the city centre and St Ives. A bus arrived and people began to board. One lady got on and showed her ticket to the driver, who said that it was not valid for this bus. She got off again, and a man who was also at the bus stop explained to her that she had a ticket for a regular bus (not a guided bus) and would have to go to the bus stop for regular buses, even to reach the same destination (the city centre). A third passenger who was waiting at the bus stop said “I thought a bus is a bus”. “Ah, but this is a guided bus” the man said, with raised eyebrows.*

In this example, not only was the busway perceived to offer an ordinary bus service with no advantages over regular systems (as another passenger said, *“I don’t see what the fuss is about”*) – but the interactions of the second and third passengers reveal the extent to which they had reflected on the service, monitoring and judging it during early experiences, and arriving at a definitive view such as *“a bus is a bus”.* There was a feeling of general annoyance amongst this group of travellers that the busway was being differentiated from other, regular buses, even though their practical experience of it was, in the end, that it was merely another bus route. Occasionally, the sense of a lack of coherence with the bus system they already knew well had a profoundly negative impact: in a similar situation to the one above, a woman who was surprised that her ticket was not valid on the busway exclaimed *“never again”*.

In stark contrast, some previous car users experienced the busway so positively that they experienced a previously unintended switch in usual practice from car to busway commuting:

*I boarded a bus at Addenbrooke’s Hospital, towards St Ives, and sat near a man who also got on there (aged around 30–40 years old). He explained that he was on the busway today to get to and from the hospital for an appointment. The first time he took the busway was when he had a hospital appointment. It was good, so he thought he’d try it to get to work. He now takes the busway to work each day (he used to commute by motorbike). Before he needed to get to the hospital, he wouldn’t have even considered or dreamed of using the bus. He said “I wanted to hate it” [the busway] because of all the bad publicity and it being late and over budget; even though he lives near the busway he was negative about it. But then when he tried it he really liked it. He said he’s “been converted”. It’s quick. And it’s comfortable. It’s better than a regular bus. He wouldn’t use other public transport – it’s unreliable. He’s told his friends how good it is – he’s “converted” more people.*

Despite this man’s initial perception of the busway, and strong lack of intention to use and like it, after “trialling” it he integrated the busway into his daily routine. The relative value and benefit (in terms of speed and comfort) for his journey to work in comparison to the car, coupled with it being coherent with his daily work schedule, led to it becoming normalized and embedded in his daily life.

## Discussion

This ethnographic account has revealed ‘micro-level’ experiences of individuals and small groups following the introduction of the Cambridgeshire Guided Busway. Our findings have implications for research and policy regarding the promotion of travel behaviour change (and its related health benefits) and for population-based interventions more broadly. We drew on some of the key elements of NPT to analyse our data and highlight the small, everyday experiences that determined how people interacted with and appraised the new transport system in the first few months of its implementation, and the ways in which people adapted and learned both individually and collectively. Rather than consider these as trivial, we contend that the summation of these apparently minor, social experiences might determine the extent to which a multi-million-pound infrastructure project is successful over time. Those introducing transport interventions of this kind should ensure that they are easy to integrate into everyday life, particularly work routines, to maximize the number of people for whom it will become normalized.

Related research conducted by our group will examine the health impacts of the busway among those who use it. Our aim in this study was not to examine those impacts, but to explore whether and how the busway became embedded in everyday practice, which is likely to be important in explaining any subsequent effects on health. We have previously reported that commuters are motivated by convenience, speed, cost and reliability when making decisions about how to travel, rather than by health considerations [36]. Similarly, the present study has shown that a complex interplay of aspects of coherence and reflexive monitoring influences travellers’ experiences and use of the busway, which may in turn have intended or unintended consequences for health because public transport is associated with greater walking and overall physical activity than car use [1] [2] [3].

We identified a clear distinction between two groups of passengers – previous car users and previous bus users – that we did not anticipate at the beginning of the research. Previous car users attributed a high value to the busway due to the perception of improved cost, convenience and comfort compared to car use. Some positively differentiated the busway from regular buses, and attributed its advantages to the innovative system in particular rather than to public transport in general, so much so that some described themselves as not actually being public transport users. This suggests that introducing systems that are perceived to be innovative and different from existing ones, rather than improving existing public transport services, may lead to a greater uptake of public transport amongst non-users.

This group was not surprised or frustrated by lack of coherence between busway and regular bus-use practices. That this group experienced and appraised the busway positively is a promising indication of the potential public health impacts of innovative public transport provision, because a shift from car use to public transport has the potential to increase population physical activity and thereby to improve health, as well as reduce greenhouse gas emissions [4] [5].

Positive first experiences of the busway led to regular use by some previous car users, despite their lack of intention to become regular users. This suggests that enabling and encouraging people to “trial” new services may lead to regular use in some who would otherwise not anticipate doing so. Previous research has demonstrated that providing car drivers with a one-month free bus ticket led to more frequent bus use and more positive attitudes towards bus travel than those observed in a control group with no free bus ticket [37]. In another study, provision of a free month travel card to car owners increased commuting by public transport up to five months later [38].

In contrast to previous car users, previous bus users tended not to attribute value and benefit to the busway over the regular bus network. Since after a very short time this group did not differentiate guided buses from regular buses, they were frustrated with the apparently arbitrary discrimination of the busway from other services (for example the requirements for different tickets, and different bus-stops in the non-guided section through the city). Although a shift from bus to guided bus may not be as significant an outcome for the innovation as that from car to guided bus, it is important to address the negative experiences of previous bus users. A more positive appraisal by this group could be achieved in two ways: making the new transport infrastructure more coherent with previous regular bus-use practices, or ensuring that the benefits of the new system outweigh those of the regular system.

In future research, both in the Commuting and Health in Cambridge study and among the wider research community, it will be important to distinguish between previous car users and previous bus users when evaluating the impacts of public transport innovations. Although both groups may arrive at the same end result – such as a shift to using the busway routinely – they are likely to reach it through different pathways (for example via positive appraisal and active choice; or via negative appraisal and a feeling of no other choice). This might have more subtle and enduring implications for everyday usage. It is important to examine not only quantitative measures of how people travel before and after interventions are implemented, but also the processes by which they are trialled and integrated (or not) into everyday practice. Furthermore, although the number of journeys made in the weeks following the introduction of the busway led local councillors to hail it as “very successful” and claim that “the people of Cambridgeshire have embraced this [busway] wholeheartedly” [39], our results indicate that by the end of the four-month study period it was still not fully normalized in the local population, and that negative opinion continued to shape people’s experiences.

The ethnographic method adopted here enabled the intervention, and experiences and attitudes towards it, to be to be examined naturalistically, in context. As our research identified, and in keeping with a core aspect of NPT, innovations are experienced differently according to existing practice. Furthermore, as our vignettes illustrate, participants engage actively and creatively with new infrastructure rather than being passive recipients. The themes underlying the critical distinction between previous bus users and previous car users would not have been uncovered in such depth by more structured methods such as surveys.

NPT was a useful conceptual framework with which to analyse and interpret our data. Different constructs of NPT were configured in different ways for the two groups. For previous bus users, coherence with the regular bus system that preceded the busway was an important construct which influenced how the busway was perceived and evaluated. Previous car users were less concerned about coherence with previous practice, but were influenced by what they perceived and understood the benefits of the busway to be over their previous practice (the reflexive monitoring domain of NPT).

There are of course limitations to our approach. The study took place in a specific context and focused on a specific intervention. By UK standards, Cambridge is relatively affluent with well-educated residents; and the Cambridgeshire Guided Busway is a unique, innovative transport system. The details of our findings may not therefore be generalizable to similar interventions in different settings, or to different interventions. Furthermore, data collection took place during autumn and winter because those were the seasons that immediately followed the opening of the busway; it is possible that travellers’ experiences and attitudes towards the busway compared to other travel modes may have varied according to the seasons. By conducting the study in the weeks following the opening of the busway we were able to observe passengers “trialling” it, some of whom would become regular users and others who would not. We were not able to observe travellers who did not trial the busway at all in this study, although related research conducted by our group will go on to compare users and non-users using other methods. Overall, our research presents an innovative set of interpretive themes that should be considered in future contexts with the purpose of refining the theoretical dimensions at the same time as testing the degree to which these elements are applicable in other contexts.

### Conclusions

This ethnographic account of the introduction of an innovative public transport system in Cambridgeshire, UK, has furthered our understanding of the processes of normalization of new interventions. Although the specific provision of a guided busway is unlikely to be widely introduced, this research highlights what users perceive to be important issues regarding new transport provision and gives insight into mechanisms of travel behaviour change. A rich qualitative account has made it possible to appreciate the varied and creative ways in which people come to learn about a new transport system and integrate it into their everyday practice. In particular, we have shown how apparently small and relatively inconsequential experiences can play a significant role in people’s initial evaluations. Our study therefore has broader implications. Public transport interventions may need to be marketed and promoted differently to different groups, for example by emphasising novelty to car users while ensuring that the new system is seen by regular bus users as being coherently integrated with existing services. Despite the temptation to herald the introduction of new transport infrastructure triumphantly, precipitating a wide range of high expectations, it may be just as important to address the ways in which public transport is a dimension of people’s routines that is valued precisely because it is embedded unproblematically in their everyday lives.
